# Origins of disparities in preventable child mortality in England and Sweden: a birth cohort study

**DOI:** 10.1136/archdischild-2018-316693

**Published:** 2019-06-26

**Authors:** Ania Zylbersztejn, Ruth Gilbert, Anders Hjern, Pia Hardelid

**Affiliations:** 1 Population, Policy and Practice, University College London Great Ormond Street Institute of Child Health, London, UK; 2 Farr Institute of Health Informatics Research, London, UK; 3 Children and Families Policy Research Unit, University College London Great Ormond Street Institute of Child Health, London, UK; 4 Centre for Health Equity Studies (CHESS), Stockholm University, Stockholm, Sweden; 5 Clinical Epidemiology Unit, Department of Medicine, Karolinska Institutet, Stockholm, Sweden

**Keywords:** child mortality, respiratory tract Infection, sudden unexpected death In infancy, England, Sweden

## Abstract

**Objective:**

To compare mortality in children aged <5 years from two causes amenable to healthcare prevention in England and Sweden: respiratory tract infection (RTI) and sudden unexpected death in infancy (SUDI).

**Design:**

Birth cohort study using linked administrative health databases from England and Sweden.

**Setting and participants:**

Singleton live births between 2003 and 2012 in England and Sweden, followed up from age 31 days until the fifth birthday, death or 31 December 2013.

**Main outcome measures:**

The main outcome measures were HR for RTI-related mortality at 31–364 days and at 1–4 years and SUDI mortality at 31–364 days in England versus Sweden estimated using Cox proportional hazards models. We calculated unadjusted HRs and HRs adjusted for birth characteristics (gestational age, birth weight, sex and congenital anomalies) and socioeconomic factors (maternal age and socioeconomic status).

**Results:**

The English cohort comprised 3 928 483 births, 768 RTI-related deaths at 31–364 days, 691 RTI-related deaths at 1–4 years and 1166 SUDIs; the corresponding figures for the Swedish cohort were 1 012 682, 131, 118 and 189. At 31–364 days, unadjusted HR for RTI-related death in England versus Sweden was 1.52 (95% CI 1.26 to 1.82). After adjusting for birth characteristics, the HR reduced to 1.16 (95% CI 0.96 to 1.40) and for socioeconomic factors to 1.11 (95% CI 0.92 to 1.34). At 1–4 years, unadjusted HR was 1.58 (95% CI 1.30 to 1.92) and decreased to 1.32 (95% CI 1.09 to 1.61) after adjusting for birth characteristics and to 1.30 (95% CI 1.07 to 1.59) after further adjustment for socioeconomic factors. For SUDI, the respective HRs were 1.59 (95% CI 1.36 to 1.85) in the unadjusted model, and 1.40 (95% CI 1.20 to 1.63) after accounting for birth characteristics and 1.19 (95% CI 1.02 to 1.39) in the fully adjusted model.

**Conclusion:**

Interventions that improve maternal health before and during pregnancy to reduce the prevalence of adverse birth characteristics and address poverty could reduce child mortality due to RTIs and SUDIs in England.

**What is already known on this topic?**

- Mortality in children aged <5 years old is nearly twice as high in England as in Sweden.
- Organisation of paediatric services and support for families with young children differ between the two countries.
- A comparison of mortality from potentially preventable causes could inform health system responses in England.

**What this study adds?**

- We compared mortality from two causes amenable to healthcare prevention using linked administrative data: respiratory tract infection (RTI) and sudden unexpected death in infancy (SUDI).
- RTI-related and SUDI mortality were 50%–60% higher in England versus Sweden; these differences were largely explained by England’s high prevalence of adverse birth characteristics.
- Interventions focusing on maternal health and well-being to improve children’s health at birth could lead to reductions in preventable mortality in England versus Sweden.

## Introduction

Mortality in children aged <5 years old in England is nearly twice as high as in Sweden,^1^ despite the two countries having comparable levels of economic development and universal healthcare. These differences can be largely attributed to increased prevalence of adverse birth characteristics (such as preterm birth or presence of congenital anomalies) in England.^2^ However, organisation of child healthcare services and support for families with young children also differ between the two countries. For example, in Sweden preventive child health services are provided in nurse-led child health centres rather than by general practitioners and health visitors in primary care,^3 4^ paid parental leave is longer,^5^ and costs of day care are heavily subsidised.^6^ A comparison of mortality from potentially preventable causes could serve as a natural experiment of the differing policy contexts and inform health system responses in England.

We compared child mortality from two potentially preventable causes of death: respiratory tract infection (RTI) and unexplained sudden unexpected death in infancy (SUDI). RTIs are a leading cause of hospital admissions among children in England and Sweden^7 8^; RTI-related deaths are considered to be amenable to healthcare intervention through vaccination (particularly for high-risk groups) and timely treatment with antibiotics.^9^ Sudden infant death syndrome (SIDS, defined as an unexpected infant death that remains unexplained following a complete death investigation^10^) is also amenable to preventive public health measures, such as advice on safe sleeping practices or smoking cessation programmes.^11^ However, comparisons of SIDS mortality are susceptible to bias due to intercountry differences in child death investigation practices.^12^ Instead, we focus on a broader grouping of causes of death—unexplained SUDI—recommended for international comparisons, which includes all unexpected deaths in infancy (ie, SIDS, deaths from unascertained causes, and explained but sudden and unexpected deaths such as those due to accidental asphyxia).

We compared RTI-related and SUDI mortality in England and Sweden using birth cohorts from linked administrative health databases. In order to inform policy makers in England as to which preventive strategies would most effectively reduce RTI-related and SUDI mortality, we need to disentangle the contribution of risk factors operating before and during pregnancy and risk factors operating after birth, whether in the healthcare setting or at home. Therefore, we determined how much of the excess preventable child mortality in England is explained by intercountry differences in the distribution of birth characteristics (as indicators of health and well-being of mothers before and during pregnancy), socioeconomic factors operating after birth and how much was due to other factors operating after birth.

## Methods

### Study population

We derived nationally representative cohorts of singleton live births in 2003–2012 to resident mothers using linked administrative databases from England and Sweden. The English birth cohort was based on birth admissions identified in Hospital Episode Statistics (HES).^13^ For each birth, two new records are created in HES—a birth admission for a baby and a delivery admission for the mother, both covering information about key risk factors at birth. The two records were probabilistically linked to enhance completeness of birth characteristics and socioeconomic factors in the birth cohort.^14^ Birth records were then linked to the baby’s subsequent hospital admissions and Office for National Statistics death registration records using a study-specific identifier generated by the data provider.^13^ We excluded 29% of births in hospitals with low completeness of recorded variables or poor linkage to mortality records. Detailed derivation and validation of the cohort is described elsewhere.^2^ The Swedish birth cohort was based on births identified in the Medical Birth Register.^15^ Births were linked to hospital records^16^ and death records^17^ using the child’s pseudonymised personal identity number generated by the data provider.

Children were followed up from the 31st day of life until their fifth birthday, death or 31 December 2013, whichever occurred first. We excluded deaths at ≤27 days due to differences in death certification practices in England and Sweden.^18 19^ Deaths at 28–30 days were excluded as 75% of these deaths in the English birth cohort were missing all causes of death, which likely reflects a data extraction error by the data provider.

### Outcomes

RTI-related deaths were defined as non-injury, non-SUDI with an appropriate International Statistical Classification of Diseases and Related Health Problems 10th Revision (ICD-10) code recoded as any cause of death or any diagnosis in a hospital admission that started <31 days before death (listed in online supplementary appendix table 1).^9^ We excluded injury-related deaths as they are considered accidental and not a direct result of RTI. Deaths were classified as injury-related if the underlying cause of death included any code from chapter 20 of ICD-10.^20^

10.1136/archdischild-2018-316693.supp1 Supplementary data

SUDI was indicated if the underlying cause of death included any ICD-10 code from a code list recommended for international comparisons to minimise bias from differences in coding and investigative practices (listed in online supplementary appendix table 2).^12^ The code list included all unexpected deaths: SIDS, sudden deaths from unspecified causes and sudden deaths that were subsequently explained (eg, accidental suffocation).

### Risk factors

We categorised birth weight as 500–1499 g, 1500–2499 g, 2500–3499 g and ≥3500 g and gestational age as 24–34, 35–36, 37–38 and ≥39 weeks. We developed an indicator of congenital anomalies using a subgroup of codes from chapter 17 of ICD-10^20^ included in a code list of chronic conditions in children.^9^ Congenital anomalies were identified if any diagnosis in hospital admissions within the first 2 years of life or any cause of death under the age of 5 included a relevant ICD-10 code.

Measures of socioeconomic status (SES) were not directly comparable in the two countries. In England, quintiles of SES were calculated using the Index of Multiple Deprivation score, an area-level indicator measured per 200–1400 households, allocated according to a child’s postcode.^13^ In Sweden, we used quintiles of family’s disposable household income in the year before childbirth.^21^ We also included maternal age as an individual-level variable indicating SES, categorised as <20, 20–24, 25–29, 30–34 and ≥35 years.

We excluded births with missing information on either of the risk factors and births weighing <500 g or at <24 weeks’ gestation.

### Statistical analyses

For each country we derived numbers and percentages of all children, total deaths, RTI-related deaths and SUDIs by each risk factor category. We also calculated cause-specific mortality rates per 100 000 child-years. We fitted Cox proportional hazards (PH) regression models to estimate HRs for cause-specific mortality in England relative to Sweden (the baseline), which we used as a measure of relative risk of death in England versus Sweden. First, we fitted unadjusted models including only a covariate for country of birth. Then, we added birth characteristics and socioeconomic factors to determine the contribution of these risk factors to excess mortality in England.

We used Schoenfeld residual plots for each covariate to test the underlying assumption of the Cox PH models that HRs for all covariates remain constant over time.^22^ We fitted additional Cox PH regression models with an effect modification term between each covariate for which PH assumption was violated and survival time.

Since 90% of RTI-related child deaths in England occur among children with at least one chronic condition, we repeated the analyses for RTI-related mortality at 1–4 years also including an indicator of chronic conditions (identified if any diagnosis recorded during infancy included ICD-10 codes from the code list of chronic conditions, excluding codes for congenital anomalies).^9^ We did not repeat the analyses for RTI-related mortality at 31–364 days as any chronic illness at age 0–30 days was likely to be associated with birth characteristics already included in the model. We used Stata MP V.14.2 for all analyses.

## Results

### Study population

**Figure 1:**
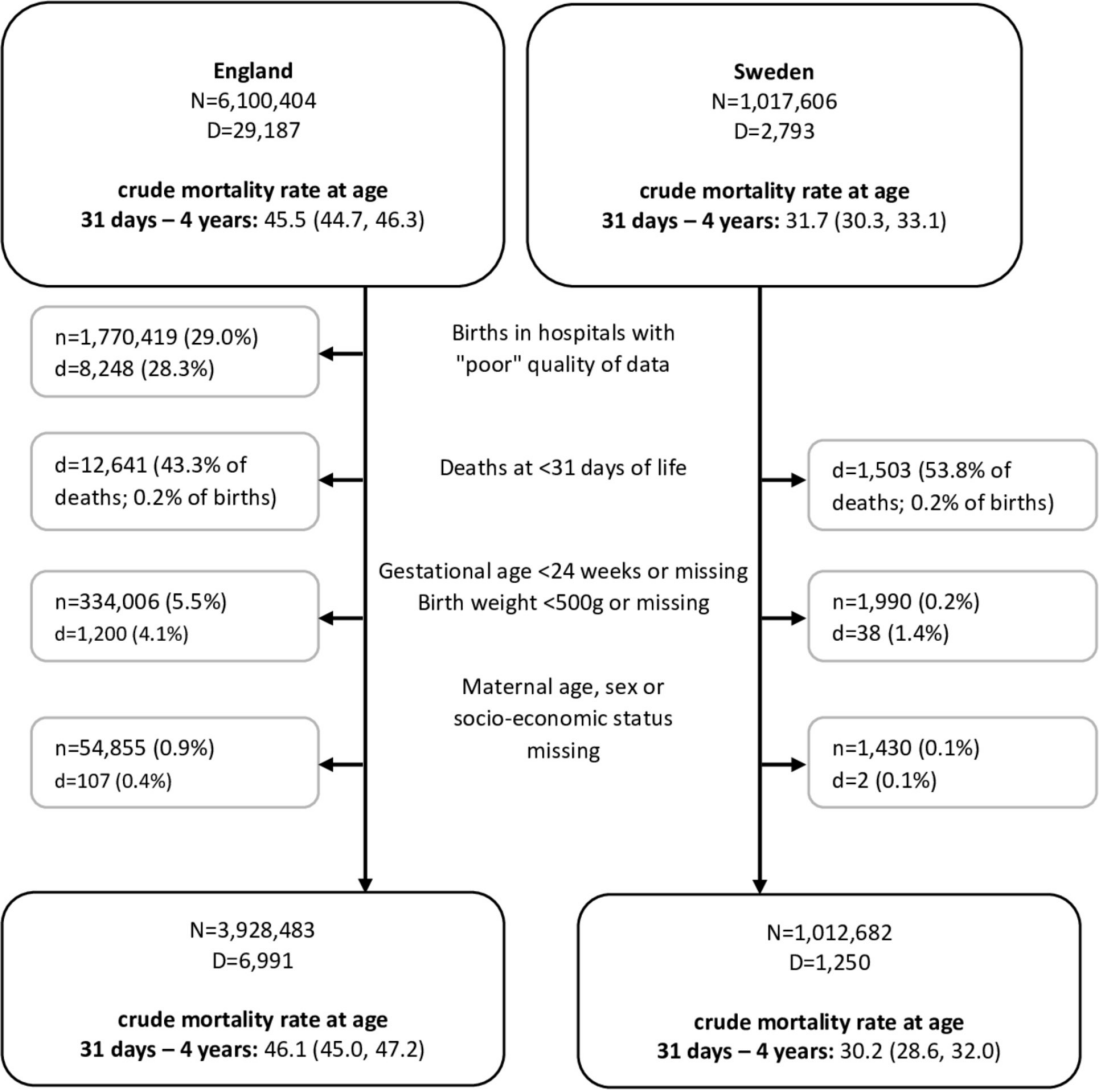
Flow diagram showing steps taken to develop comparable, nationally representative cohorts of children who were born in 2003–2012 in England and Sweden. The numbers of live births (n) and deaths (d) are presented. For each exclusion criterion, the percentage of all live births and all deaths is shown in brackets. Crude mortality rates at age 31 days–4 years per 100 000 child-years are presented for each country before and after applying all exclusion criteria (95% CI).

The cohort comprised 3 928 483 births in England and 1 012 682 births in Sweden, with follow-up of 15 167 185 and 4 132 324 child-years, respectively (figure 1). The prevalence of adverse birth characteristics such as low birth weight (<2500 g), preterm birth (at <37 weeks’ gestation) and congenital anomalies was higher in England than in Sweden (table 1). Young motherhood (aged <20 years) was also more common in England.

**Table 1:** Characteristics of children who survived beyond 30 days of life and children who died (by cause of death and age at death) in England and Sweden

	Live births		All deaths at 31–364 days		All deaths at 1–4 years		RTI-related deaths at 31–364 days		RTI-related deaths at 1–4 years		SUDIs at 31–364 days	
	England (n=3 928 483)	Sweden (n=1 012 682)	England (n=4768)	Sweden (n=774)	England (n=2223)	Sweden (n=476)	England (n=768)	Sweden (n=131)	England (n=691)	Sweden (n=118)	England (n=1166)	Sweden (n=189)
Birth weight (g)												
<1500	26 221 (0.67)	4646 (0.46)	863 (18)	112 (14)	107 (4.8)	14 (2.9)	76 (9.9)	13 (9.9)	37 (5.4)	6 (5.1)	53 (4.5)	17 (9)
1500–2499	189 481 (4.8)	25 674 (2.5)	918 (19)	126 (16)	306 (14)	47 (9.9)	172 (22)	30 (23)	109 (16)	18 (15)	197 (17)	15 (7.9)
2500–3499	2 089 095 (53)	428 894 (42)	2198 (46)	332 (43)	1217 (55)	216 (45)	396 (52)	49 (37)	387 (56)	52 (44)	651 (56)	92 (49)
≥3500	1 623 686 (41)	553 468 (55)	789 (17)	204 (26)	593 (27)	199 (42)	124 (16)	39 (30)	158 (23)	42 (36)	265 (23)	65 (34)
Gestational age (weeks)												
24–34	85 340 (2.2)	17 616 (1.7)	1155 (24)	158 (20)	169 (7.6)	34 (7.1)	133 (17)	25 (19)	55 (8)	13 (11)	119 (10)	22 (12)
35–36	136 681 (3.5)	30 220 (3)	433 (9.1)	77 (9.9)	144 (6.5)	28 (5.9)	75 (9.8)	10 (7.6)	51 (7.4)	11 (9.3)	116 (9.9)	16 (8.5)
37–38	726 161 (18)	191 005 (19)	1088 (23)	182 (24)	516 (23)	101 (21)	183 (24)	33 (25)	184 (27)	23 (19)	291 (25)	45 (24)
≥39	2 980 301 (76)	773 841 (76)	2092 (44)	357 (46)	1394 (63)	313 (66)	377 (49)	63 (48)	401 (58)	71 (60)	640 (55)	106 (56)
Baby’s sex												
Boy	2 014 198 (51)	520 597 (51)	2731 (57)	437 (56)	1202 (54)	260 (55)	416 (54)	76 (58)	367 (53)	66 (56)	734 (63)	117 (62)
Girl	1 914 285 (49)	492 085 (49)	2037 (43)	337 (44)	1021 (46)	216 (45)	352 (46)	55 (42)	324 (47)	52 (44)	432 (37)	72 (38)
Congenital anomalies												
No	3 815 315 (97)	988 298 (98)	2626 (55)	456 (59)	1386 (62)	358 (75)	370 (48)	72 (55)	347 (50)	75 (64)	1117 (96)	165 (87)
Yes	113 168 (2.9)	24 384 (2.4)	2142 (45)	318 (41)	837 (38)	118 (25)	398 (52)	59 (45)	344 (50)	43 (36)	49 (4.2)	24 (13)
Chronic condition (diagnosed throughout infancy)												
No	3 753 493 (96)	985 057 (97)			1132 (51)	265 (56)			241 (35)	51 (43)		
Yes	174 990 (4.5)	27 625 (2.7)			1091 (49)	221 (44)			450 (65)	67 (57)		
Maternal age (years)												
<20	241 111 (6.1)	16 149 (1.6)	552 (12)	34 (4.4)	189 (8.5)	16 (3.4)	70 (9.1)	8 (6.1)	51 (7.4)	3 (2.5)	222 (19)	12 (6.3)
20–24	757 667 (19)	129 130 (13)	1152 (24)	145 (19)	553 (25)	73 (15)	179 (23)	28 (21)	163 (24)	16 (14)	358 (31)	48 (25)
25–29	1 063 293 (27)	295 730 (29)	1209 (25)	222 (29)	571 (26)	131 (28)	210 (27)	42 (32)	178 (26)	32 (27)	282 (24)	55 (29)
30–34	1 109 174 (28)	356 128 (35)	1063 (22)	212 (27)	555 (25)	156 (33)	168 (22)	31 (24)	187 (27)	45 (38)	176 (15)	44 (23)
≥35	757 238 (19)	215 545 (21)	792 (17)	161 (21)	355 (16)	100 (21)	141 (18)	22 (17)	112 (16)	22 (19)	128 (11)	30 (16)
Quintile of socioeconomic status												
Q1: most deprived	851 119 (22)	201 434 (20)	1534 (32)	239 (31)	627 (28)	115 (24)	247 (32)	51 (39)	201 (29)	29 (25)	393 (34)	69 (37)
Q2	803 435 (20)	200 305 (20)	1185 (25)	162 (21)	489 (22)	114 (24)	197 (26)	27 (21)	145 (21)	23 (19)	315 (27)	42 (22)
Q3	767 678 (20)	202 580 (20)	813 (17)	105 (14)	422 (19)	94 (20)	132 (17)	13 (9.9)	126 (18)	27 (23)	209 (18)	22 (12)
Q4	762 378 (19)	204 117 (20)	700 (15)	127 (16)	366 (16)	85 (18)	110 (14)	20 (15)	108 (16)	20 (17)	162 (14)	26 (14)
Q5: least deprived	743 873 (19)	204 246 (20)	536 (11)	141 (18)	319 (14)	68 (14)	82 (11)	20 (15)	111 (16)	19 (16)	87 (7.5)	30 (16)

Data are number (%) of children who survived beyond 30 days of life, of all deaths, of RTI-related deaths at 31–364 days and at 1–4 years, and of SUDIs at 31–364 days. Column total may not add up to 100% due to rounding.

RTI, respiratory tract infection; SUDI, sudden unexpected death in infancy.

RTI-related deaths contributed 16% (768) of all deaths at 31–364 days in England and 17% (131) in Sweden. In both countries, a third of children who died in the first year of life with RTI had low birth weight, 27% were born prematurely, half had a congenital anomaly (table 1), and over 70% had at least one of these risk factors (559 in England and 92 in Sweden). At 1–4 years, RTI-related deaths contributed 31% (691) of all deaths in England and 25% (118) in Sweden, of which approximately 20% were in children with low birth weight or preterm birth, 50% and 36%, respectively, were in children with congenital anomalies, and 69% and 57%, respectively, were in children with any of these risk factors (475 in England and 67 in Sweden). In both countries SUDI accounted for a further 24% of all deaths at 31–364 days (1166 in England and 189 in Sweden). One in five of these deaths occurred in children with low birth weight or preterm birth, 4% in England and 13% in Sweden in children with congenital anomalies, and half in children with any of these characteristics (565 in England and 93 in Sweden). Unadjusted mortality rates were 50%–60% higher in England than in Sweden (online supplementary appendix table 3).

### Comparison of RTI-related and SUDI mortality

At 31–364 days, children born in England had 52% higher risk of RTI-related death than children born in Sweden (HR=1.52, 95% CI 1.26 to 1.82; table 2). After adjusting for birth characteristics, the relative risk of RTI-related death in England versus Sweden reduced by approximately 70%, to 16% higher relative risk (HR=1.16, 95% CI 0.96 to 1.40). Further adjustment for socioeconomic factors reduced the relative risk to 11% (HR=1.11, 95% CI 0.92 to 1.34). The risk of RTI-related death was highest for children with congenital anomalies (HR=25.55, 95% CI 22.20 to 29.41, compared with children with no anomalies) and low birth weight (HR=5.30, 95% CI 3.54 to 7.94 for 500–1499 g and HR=5.38, 95% CI 4.13 to 7.02 for 1500–2499 g vs birth weight ≥3500 g).

**Table 2:** Unadjusted and adjusted Cox PH regression models for RTI-related mortality at 31–364 days in England relative to Sweden in 2003–2012

	Model 1	Model 2	Model 3
Country			
England	1.52 (1.26 to 1.82)	1.16 (0.96 to 1.40)	1.11 (0.92 to 1.34)
Sweden (baseline)	1	1	1
Birth weight (g)			
500–1499		5.94 (3.97 to 8.89)	5.30 (3.54 to 7.94)
1500–2499		5.98 (4.59 to 7.79)	5.38 (4.13 to 7.02)
2500–3499		2.11 (1.75 to 2.53)	2.00 (1.66 to 2.41)
≥3500 (baseline)		1	1
Gestational age (weeks)			
24–34		1.18 (0.86 to 1.61)	1.23 (0.90 to 1.68)
35–36		1.29 (0.98 to 1.68)	1.32 (1.01 to 1.72)
37–38		1.20 (1.01 to 1.43)	1.21 (1.02 to 1.44)
≥39 (baseline)		1	1
Sex			
Boy		1.05 (0.92 to 1.20)	1.04 (0.91 to 1.19)
Girl (baseline)		1	1
Congenital anomaly			
Yes		25.72 (22.34 to 29.61)	25.55 (22.20 to 29.41)
No (baseline)		1	1
Maternal age (years)			
<25			1.34 (1.12 to 1.62)
25–29			1.24 (1.03 to 1.49)
30–34 (baseline)			1
≥35			1.14 (0.93 to 1.40)
Quintile of socioeconomic status			
Q1: most deprived			1.96 (1.56 to 2.47)
Q2			1.73 (1.36 to 2.19)
Q3			1.25 (0.97 to 1.62)
Q4			1.19 (0.91 to 1.54)
Q5: least deprived (baseline)			1

Data are adjusted HR with 95% CI. Each column represents a separate Cox PH model: model 1 was only adjusted for indicator of country with Sweden as baseline, model 2 was additionally adjusted for birth characteristics (birth weight, gestational age and presence of congenital anomalies), and model 3 was further adjusted for socioeconomic factors (socioeconomic status and maternal age).

PH, proportional hazards; RTI, respiratory tract infection.

At 1–4 years, the risk of RTI-related death was 58% higher in England relative to Sweden (HR=1.58, 95% CI 1.30 to 1.92; table 3). After adjusting for birth characteristics, the relative risk of RTI-related death in England versus Sweden nearly halved to 32% higher risk (HR=1.32, 95% CI 1.09 to 1.61) and did not change appreciably after further adjustment for socioeconomic factors. Having a congenital anomaly increased the risk of RTI-related death 28 times relative to having no anomalies (HR=28.39, 95% CI 24.55 to  32.82).

**Table 3:** Unadjusted and adjusted Cox PH models for RTI-related mortality at 1–4 years in England relative to Sweden in 2003–2012

	Model 1	Model 2	Model 3
Country			
England	1.58 (1.30 to 1.92)	1.32 (1.09 to 1.61)	1.30 (1.07 to 1.59)
Sweden (baseline)	1	1	1
Birth weight (g)			
500–1499		3.12 (2.02 to 4.83)	2.99 (1.93 to 4.63)
1500–2499		3.67 (2.78 to 4.84)	3.50 (2.65 to 4.62)
2500–3499		1.70 (1.43 to 2.02)	1.66 (1.39 to 1.97)
≥3500 (baseline)		1	1
Gestational age (weeks)			
<37		0.85 (0.64 to 1.13)	0.86 (0.65 to 1.15)
37–38		1.18 (1.00 to 1.41)	1.20 (1.01 to 1.42)
≥39 (baseline)		1	1
Sex			
Boy		0.99 (0.86 to 1.13)	0.98 (0.85 to 1.13)
Girl (baseline)		1	1
Congenital anomaly			
Yes		28.42 (24.58 to 32.86)	28.39 (24.55 to 32.82)
No (baseline)		1	1
Maternal age (years)			
<25			1.04 (0.86 to 1.25)
25–29			0.92 (0.76 to 1.11)
30–34 (baseline)			1
≥35			0.81 (0.66 to 1.00)
Quintile of socioeconomic status			
Q1: most deprived			1.28 (1.02 to 1.60)
Q2			1.08 (0.85 to 1.36)
Q3			1.07 (0.84 to 1.35)
Q4			0.93 (0.73 to 1.19)
Q5: least deprived (baseline)			1

Data are adjusted HR with 95% CI. Each column represents a separate Cox PH model: model 1 was only adjusted for indicator of country with Sweden as baseline, model 2 was additionally adjusted for birth characteristics (birth weight, gestational age and presence of congenital anomalies), and model 3 was further adjusted for socioeconomic factors (socioeconomic status and maternal age).

PH, proportional hazards; RTI, respiratory tract infection.

Children born in England had 59% higher risk of SUDI than children born in Sweden (HR=1.59, 95% CI 1.36 to 1.85; table 4). Adjustment for birth characteristics and socioeconomic factors each reduced the relative risk of SUDI in England versus Sweden by a third, to 40% (HR=1.40, 95% CI 1.20 to 1.63) and 19% (HR=1.19, 95% CI 1.02 to 1.39), respectively. The risk of SUDI was highest for children with low birth weight (HR=6.83, 95% CI 4.62 to 10.09 for 500–1499 g and HR=3.33, 95% CI 2.66 to 4.18 for 1500–2499 g vs birth weight ≥3500 g) and for children of young mothers (HR=4.45, 95% CI 3.86 to 5.37 for mothers aged <20 years vs 30–34 years).

**Table 4:** Unadjusted and adjusted Cox PH models for mortality from SUDI at 31–364 days in England relative to Sweden in 2003–2012

	Model 1	Model 2	Model 3
Country			
England	1.59 (1.36 to 1.85)	1.40 (1.20 to 1.63)	1.19 (1.02 to 1.39)
Sweden (baseline)	1	1	1
Birth weight (g)			
500–1499		8.58 (5.81 to 12.67)	6.83 (4.62 to 10.09)
1500–2499		4.17 (3.33 to 5.22)	3.33 (2.66 to 4.18)
2500–3499		1.80 (1.57 to 2.06)	1.59 (1.39 to 1.83)
≥3500 (baseline)		1	1
Gestational age (weeks)			
24–34		1.81 (1.35 to 2.43)	1.98 (1.48 to 2.66)
35–36		2.12 (1.71 to 2.63)	2.25 (1.82 to 2.79)
37–38		1.44 (1.26 to 1.65)	1.52 (1.33 to 1.74)
≥39 (baseline)		1	1
Sex			
Boy		1.68 (1.51 to 1.88)	1.66 (1.49 to 1.86)
Girl (baseline)		1	1
Congenital anomaly			
Yes		1.09 (0.85 to 1.40)	1.07 (0.83 to 1.37)
No		1	1
Maternal age (years)			
<20			4.45 (3.68 to 5.37)
20–24			2.45 (2.08 to 2.90)
25–29			1.49 (1.26 to 1.77)
30–34 (baseline)			1
≥35			1.06 (0.86 to 1.30)
Quintile of socioeconomic status			
Q1: most deprived			2.19 (1.78 to 2.70)
Q2			2.05 (1.66 to 2.53)
Q3			1.54 (1.23 to 1.93)
Q4			1.41 (1.12 to 1.78)
Q5: least deprived (baseline)			1

Data are adjusted HR with 95% CI. Each column represents a separate Cox PH model: model 1 was only adjusted for indicator of country with Sweden as baseline, model 2 was additionally adjusted for birth characteristics (birth weight, gestational age and presence of congenital anomalies), and model 3 was further adjusted for socioeconomic factors (socioeconomic status and maternal age).

PH, proportional hazards; SUDI, sudden unexpected death in infancy.

### Sensitivity analyses

The PH assumption was not met only for the indicator of congenital anomaly for RTI-related deaths at 31–364 days. We repeated the analyses allowing for different HRs for congenital anomaly at 1–2 months, 2–3 months and 3–12 months. The HRs did not change appreciably in sensitivity analyses (online supplementary appendix table 4). Cox PH models for RTI-related mortality at 1–4 years and SUDI met the PH assumption.

The relative risk of an RTI-related death at 1–4 years declined from 58% to 32% after adjustment for birth characteristics in the main analyses, and further to 3% after including an indicator of chronic conditions in sensitivity analyses (online supplementary appendix table 5). The relative risk did not change appreciably after further adjustment for socioeconomic factors. Children with chronic conditions had 20 times higher risk of RTI-related death compared with children with no chronic conditions; children with congenital anomalies had nearly 9 times higher risk (compared with 28 times higher risk in the main analyses without adjusting for chronic conditions).

Use of ICD-10 codes included in the definition of SUDI varied between the two countries (online supplementary appendix table 6). ICD-10 code ‘R95’ denoting SIDS accounted for 63% of SUDIs in England and 75% in Sweden; ‘R99’ for unknown cause accounted for a further 33% and 22%, respectively.

## Discussion

We compared mortality from two potentially preventable causes which together accounted for over one-third of child deaths beyond the first month of life in England and Sweden. RTI-related and SUDI mortality rates were 50%–60% higher in England than in Sweden. Differences in the distribution of birth characteristics accounted for nearly 70% reduction in the relative risk of RTI-related death at 31–364 days, 45% reduction at 1–4 years and a third of excess SUDI mortality in England relative to Sweden. Socioeconomic factors independently explained a further 10% of the excess risk of RTI-related death at 31–364 days and a third of the excess risk of SUDI in England relative to Sweden (over and above their effect on birth characteristics). Differences in the prevalence of chronic conditions likely contributed to excess RTI-related mortality beyond infancy in England.

The main strength of this study was the use of large nationally representative birth cohorts with individual-level information, which enabled us to compare specific causes of death adjusted for birth characteristics and socioeconomic factors, overcoming the limitations of previous comparisons based on official national statistics aggregated by the underlying cause of death.^23 24^ We also used broad definitions of causes of death to minimise bias due to intercountry differences in death certification practices.

Due to high rates of missing data on key birth characteristics in the English cohort, we had to exclude one-third of births in England from the analyses. The subcohort of births, however, was thoroughly validated (details reported elsewhere^2^). We were also not able to compare mortality in the first month of life, which accounts for over half of all under-5 deaths (in 2003–2012, 60% of all childhood deaths in England and Wales,^25^ and 53% in Sweden occurred at age 0–27 days). Excluding these early deaths likely underestimated the effect of adverse birth outcomes such as preterm birth or presence of congenital anomalies, which are strongly associated with increased risk of neonatal mortality.^2^ The distribution of SUDI by age at death also differed between the two countries—in Sweden, 35% of SUDIs occurred in the first month of life, compared with 18% reported for England and Wales.^26^ Further work is needed to determine whether these differences reflect variation in coding practices or better prevention of SUDIs beyond the first month of life in Sweden. We were also not able to include deaths on days 28–30 in the analyses, traditionally included when reporting postneonatal mortality rates, as a high proportion of these deaths in England did not have any recorded causes of death. However, these deaths accounted for only 4% of deaths at 28–364 days in both countries. Lastly, we did not have comparable measures of SES—we used an area-level measure in England and an individual-level measure in Sweden. The area-level indicator in England likely underestimated the true differences between SES quintiles, as individuals in a given area were given the same score, averaging local differences and reducing variation in SES scores. English national birth cohort overcoming these limitations, with near 100% completeness of risk factors at birth, high quality of linkage to mortality data and individual-level measure of SES, could be developed by linking Office for National Statistics birth registration, National Health Service birth notification data and HES records for mothers and babies.^27^ However, these data sets are not routinely linked.

### Interpretation

Previous comparisons of cause-specific child mortality hypothesised that increased infection-related mortality in England relative to Sweden reflected differences in provision and organisation of healthcare in the two countries.^23 24^ Our results suggest that risk factors operating before and during pregnancy are more important: excess RTI-related mortality in England relative to Sweden was largely explained by higher prevalence of adverse birth characteristics in England, with congenital anomalies and low birth weight being associated with the highest relative risks of RTI-related deaths. Unfavourable distribution of birth characteristics also contributed to excess SUDI mortality in England. Adverse birth characteristics are strongly associated with maternal risk factors such as smoking,^28 29^ obesity,^30 31^ and young and old maternal age,^32–34^ many of which are more prevalent in England than in Sweden. These maternal risk factors are, in turn, strongly associated with socioeconomic disadvantage.^35 36^ These findings suggest that reducing adverse maternal health and socioeconomic circumstances before and during pregnancy to improve health outcomes at birth could reduce preventable child mortality in England relative to Sweden. Further work is needed to identify specific interventions which would be most effective at improving birth characteristics of children in England.

Socioeconomic factors contributed a third of the excess risk of SUDI and 10% of the excess risk of RTI-related infant death in England relative to Sweden, over and above their effect on birth characteristics. This likely reflects wider income inequality in England relative to Sweden (in 2009, 5.5% of children in the UK lived in deprived households, compared with 1.3% in Sweden).^37^ Linkage to external data sources is needed to identify specific risk factors through which socioeconomic circumstances after birth contribute to the risk of RTI-related death or SUDI. Detailed characteristics of children who died and their familial and social environment could be described using the National Child Mortality Database (NCMD), a national data set collated from child death overview panels, available from April 2019.^38^ Additional linkage of NCMD to data on exposures in children who did not die, however, would be required to identify risk factors for child deaths.^39^ Linkage to census records in England could provide insights into the importance of factors such as the number of household members and rooms as indicators of overcrowding and housing conditions; socioeconomic inequalities in access to and uptake of preventive services could be explored through linkage to primary care records. Further analyses using causal mediation methods are also needed to quantify the total effect of socioeconomic factors on the excess cause-specific mortality in England relative to Sweden, including the effect mediated by adverse birth characteristics.

Some of the differences in RTI-related and SUDI mortality which remained unexplained could reflect differences in provision of preventive healthcare. Support for families with young children differs between the two countries; for example, in Sweden parents have longer paid parental leave and subsidised day care.^5^ Parents also build a trusting relationship with their nurse from the child health centre, who sees the family on average 14 times in the first 18 months of life.^40^ Such provision appears to improve uptake of vaccines and other preventive services.^3^ In England services are more fragmented: health visitors have only five mandated contacts with families in the first 2 years of a child’s life and some of the care is provided by general practitioners in primary care.^41^ Linkage to primary care records is needed to explore the contribution of differences in healthcare organisation and use to higher preventable child mortality in England compared with Sweden. Such data, however, are currently not collected in Sweden.

Our sensitivity analyses showed that chronic conditions contributed to an increased risk of RTI-related mortality beyond infancy in England, which remained after accounting for birth characteristics. Further work is needed to determine whether this is due to intercountry differences in the prevalence of chronic conditions, in the provision of healthcare for children with chronic conditions or in the recording of these conditions in hospitalisation records. Further comparison should also include information on what proportion of these deaths were expected (ie, whether RTI was part of terminal decline in child’s health, not possible to prevent) and what proportion were unexpected^42^.

### Policy implications

Our results suggest that policies focusing on improving the health of women and socioeconomic circumstances of their families before and during pregnancy to reduce the prevalence of maternal risk factors and in turn of adverse birth characteristics could lead to substantial reductions in RTI-related and SUDI mortality in England. After accounting for birth characteristics, socioeconomic factors and chronic illness, we found weak evidence for an excess of RTI-related and SUDI mortality in England relative to Sweden. This finding suggests that improving the socioeconomic conditions of disadvantaged families and investment in health care before, during and after pregnancy for women and their children could reduce the gap in preventable child mortality in England and Sweden.
