# Supplementary material for: Origins of disparities in preventable child mortality in England and Sweden: a birth cohort study
Source: Arch Dis Child. 2019 Jun 26;105(1):53–61. doi: 10.1136/archdischild-2018-316693 (PMC6951233; doi:10.1136/archdischild-2018-316693)
Supplement: Supplementary data [file archdischild-2018-316693supp001.pdf]

Supplementary materials

ICD-10 codes used to define outcomes

Appendix table 1– ICD-10 codes proposed by Hardelid *et al.* [1] to capture RTI-related deaths.

ICD-10 code	Definition
A15	Respiratory tuberculosis, bacteriologically and histologically confirmed
A16	Respiratory tuberculosis, not confirmed bacteriologically or histologically
A19	Miliary tuberculosis
B97.4	Respiratory syncytial virus as the cause of diseases classified to other chapters
A37	Whooping cough
J00-J06	Acute upper respiratory infections
J09-J18	Influenza and pneumonia
J20-22	Other acute lower respiratory infections

ICD-10=International Statistical Classification of Diseases and Related Health Problems 10th Revision; RTI=respiratory tract infection.

Appendix table 2 – ICD-10 codes proposed by Taylor *et al.*[2] to capture SUDI deaths.

ICD-10 code	Definition
R95	Sudden infant death syndrome
R96	Other sudden death, cause unknown
R98	Unattended death
R99	Other ill-defined and unspecified causes of mortality
W75	Accidental suffocation and strangulation in bed
W78	Inhalation of gastric contents
W79	Inhalation and ingestion of food causing obstruction of respiratory tract

ICD-10=International Statistical Classification of Diseases and Related Health Problems 10th Revision; SUDI=sudden unexpected deaths in infancy.

Additional results

Appendix table 3– Unadjusted RTI-related mortality at 31-364 days and 1-4 years and SUDI mortality at 31-364 days by birth characteristics and socio-economic factors in England and Sweden in 2003-2012

	**RTI-related deaths at 31-364 days**		**RTI-related deaths at 1-4 years**		**SUDI deaths at 31-364 days**	
**Country**	**England**	**Sweden**	**England**	**Sweden**	**England**	**Sweden**
**Total**	21 (20, 23)	14 (12, 17)	6.0 (5.5, 6.4)	3.7 (3.1, 4.4)	32 (31, 34)	20 (18, 24)
**Birth weight (g)**						
500-1499	330 (260, 410)	310 (180, 540)	49 (35, 67)	41 (18, 92)	230 (170, 300)	410 (250, 650)
1500-2499	99 (86, 120)	130 (90, 180)	19 (16, 23)	22 (14, 35)	110 (99, 130)	64 (39, 110)
2500-3499	21 (19, 23)	12 (9.4, 17)	6.3 (5.7, 6.9)	3.8 (2.9, 5.0)	34 (32, 37)	23 (19, 29)
≥3500	8.3 (7, 9.9)	7.7 (5.6, 11)	3.3 (2.8, 3.9)	2.4 (1.8, 3.2)	18 (16, 20)	13 (10, 16)
**Gestational age (weeks)**						
24-34	170 (150, 200)	160 (110, 230)	22 (17, 28)	23 (14, 40)	150 (130, 180)	140 (90, 210)
35-36	60 (48, 75)	36 (19, 67)	13 (9.5, 16)	11 (6.4, 21)	93 (77, 110)	58 (35, 95)
37-38	28 (24, 32)	19 (13, 27)	8.5 (7.4, 9.9)	3.8 (2.5, 5.7)	44 (39, 49)	26 (19, 34)
≥39	14 (13, 15)	8.9 (6.9, 11)	4.6 (4.2, 5.0)	2.9 (2.3, 3.7)	23 (22, 25)	15 (12, 18)
**Sex**						
Boy	23 (21, 25)	16 (13, 20)	6.2 (5.6, 6.9)	4 (3.1, 5.1)	40 (37, 43)	25 (20, 29)
Girl	20 (18, 22)	12 (9.4, 16)	5.7 (5.1, 6.4)	3.3 (2.5, 4.4)	25 (22, 27)	16 (13, 20)
**Congenital anomalies**						
Yes	11 (9.6, 12)	8 (6.3, 10)	3.1 (2.8, 3.4)	2.4 (1.9, 3.0)	32 (30, 34)	18 (16, 21)
No	390 (350, 430)	270 (210, 340)	110 (98, 120)	56 (42, 76)	48 (36, 63)	110 (73, 160)
**Maternal age (years)**						
<20	32 (25, 40)	54 (27, 110)	6.8 (5.2, 9.0)	5.7 (1.9, 18)	100 (88, 110)	81 (46, 140)
20-25	26 (22, 30)	24 (16, 34)	7.2 (6.2, 8.4)	4 (2.4, 6.5)	52 (47, 57)	41 (31, 54)
25-30	22 (19, 25)	16 (11, 21)	5.7 (5.0, 6.7)	3.4 (2.4, 4.8)	29 (26, 33)	20 (16, 26)
30-35	17 (14, 19)	9.5 (6.7, 14)	5.8 (5.0, 6.6)	3.9 (2.9, 5.3)	17 (15, 20)	13 (10, 18)
≥35	20 (17, 24)	11 (7.3, 17)	5 (4.2, 6.0)	3.3 (2.1, 5.0)	18 (16, 22)	15 (11, 22)
**Quintile of socio-economic status**						
Q1: most deprived	32 (28, 36)	28 (21, 36)	7.9 (6.9, 9.1)	4.6 (3.2, 6.6)	50 (46, 56)	37 (30, 47)
Q2	27 (23, 31)	15 (10, 21)	6.1 (5.2, 7.2)	3.6 (2.4, 5.4)	43 (38, 48)	23 (17, 31)
Q3	19 (16, 22)	7 (4.1, 12)	5.6 (4.7, 6.6)	4.2 (2.9, 6.1)	30 (26, 34)	12 (7.8, 18)
Q4	16 (13, 19)	11 (6.9, 17)	4.8 (4.0, 5.8)	3.1 (2.0, 4.8)	23 (20, 27)	14 (9.5, 20)
Q5: least deprived	12 (9.7, 15)	11 (6.9, 17)	5.1 (4.3, 6.2)	2.9 (1.9, 4.6)	13 (10, 16)	16 (11, 23)

RTI=respiratory tract infections. SUDI=sudden unexpected infant deaths. Data are unadjusted mortality rates per 100,000 child-years (95% confidence interval).

Sensitivity analyses

Appendix table 4 – Unadjusted and adjusted Cox proportional hazards models for RTI-related mortality at 31-364 days in England relative to Sweden including an effect modification term with time for congenital anomaly indictor (with 95% confidence interval).

Risk factor	Model 1	Model 2	Model 3
**Country**			
England	1.52 (1.26, 1.82)	1.16 (0.96, 1.40)	1.11 (0.92, 1.34)
Sweden (baseline)	1	1	1
**Birth weight (g)**			
500-1499		5.95 (3.97, 8.91)	5.32 (3.55, 7.96)
1500-2499		5.98 (4.59, 7.79)	5.39 (4.13, 7.03)
2500-3499		2.11 (1.75, 2.53)	2.00 (1.66, 2.41)
≥3500 (baseline)		1	1
**Gestational age (weeks)**			
24-34		1.18 (0.86, 1.61)	1.23 (0.90, 1.68)
35-36		1.29 (0.98, 1.68)	1.32 (1.01, 1.72)
37-38		1.20 (1.01, 1.43)	1.21 (1.02, 1.44)
≥39 (baseline)		1	1
**Sex**			
Boy		1.05 (0.92, 1.20)	1.04 (0.91, 1.19)
Girl (baseline)		1	1
**Congenital anomaly**			
Yes: 1-2 months		13.16 (9.26, 18.70)	13.07 (9.20, 18.56)
Yes: 2-3 months		18.70 (13.07, 26.74)	18.57(12.98, 26.55)
Yes: 3-12 months		31.61 (26.81, 37.29)	31.41 (26.63, 37.04)
No		1	1
**Maternal age (years)**			
<25			1.34 (1.12, 1.62)
25-29			1.24 (1.03, 1.49)
30-34 (baseline)			1
≥35			1.14 (0.93, 1.40)
**Quintile of socio-economic status**			
Q1: Most deprived			1.96 (1.56, 2.47)
Q2			1.73 (1.36, 2.19)
Q3			1.26 (0.97, 1.62)
Q4			1.19 (0.91, 1.54)
Q5: Least deprived (baseline)			1

RTI=respiratory tract infections. Data are adjusted hazard ratios. Each column represents a separate Cox proportional hazards model: model 1 was only adjusted for indicator of country with Sweden as baseline, model 2 was additionally adjusted for birth characteristics (birth weight, gestational age, presence of congenital anomalies), model 3 was further adjusted for socio-economic factors (socio-economic status and maternal age). To meet the proportional hazards assumption we allowed for different hazard ratio for indicator of congenital anomaly at 1-2 months, 2-3 months and 3-12 months.

Appendix table 5 – Unadjusted and adjusted Cox proportional hazards models for RTI-related death at 1-4 years including an indicator for chronic conditions in England relative to Sweden (with 95% confidence intervals)

	Model 1	Model 3	Model 4
**Country**			
England	1.58 (1.30, 1.92)	1.03 (0.85, 1.26)	1.03 (0.84, 1.25)
Sweden (baseline)	1	1	1
**Birth weight (g)**			
500-1499		1.25 (0.81, 1.93)	1.23 (0.79, 1.89)
1500-2499		2.67 (2.02, 3.51)	2.59 (1.96, 3.42)
2500-3499		1.57 (1.32, 1.86)	1.55 (1.30, 1.84)
3500 (baseline)		1	1
**Gestational age (weeks)(weeks)**			
<37		0.69 (0.52, 0.91)	0.69 (0.52, 0.92)
37-38		1.08 (0.91, 1.28)	1.08 (0.91, 1.29)
≥39 (baseline)		1	1
**Sex**			
Boy		0.95 (0.83, 1.10)	0.95 (0.83, 1.09)
Girl (baseline)		1	1
**Congenital anomaly**			
Yes		8.70 (7.41, 10.22)	8.74 (7.44, 10.27)
No		1	1
**Chronic condition (in infancy)**			
Yes		20.06 (16.99, 23.68)	19.88 (16.83, 23.47)
No		1	1
**Maternal age (years)**			
<25			0.96 (0.80, 1.16)
25-29			0.91 (0.75, 1.10)
30-34 (baseline)			1
≥35			0.82 (0.66, 1.01)
**Quintile of socio-economic status**			
Q1: Most deprived			1.18 (0.94, 1.47)
Q2			1.01 (0.80, 1.27)
Q3			1.03 (0.81, 1.30)
Q4			0.92 (0.72, 1.17)
Q5: Least deprived (baseline)			1

RTI=respiratory tract infections. Data are adjusted hazard ratios. Each column represents a separate Cox proportional hazards model: model 1 was only adjusted for indicator of country with Sweden as baseline, model 2 was additionally adjusted for birth characteristics (birth weight, gestational age, presence of congenital anomalies) and indicator of presence of chronic conditions, model 3 was further adjusted for socio-economic factors (socio-economic status and maternal age).

Appendix table 6 – Proportion of SUDI deaths identified using each ICD-10 code in England and in Sweden

ICD-10 code	England	Sweden
R95	63%	75%
R96	0.1%	0
R98	0	0.5%
R99	33%	22%
W75	3.5%	2.1%
W78	0.8%	0
W79	0.3%	1.1%

ICD-10=International Statistical Classification of Diseases and Related Health Problems, SUDI=sudden unexpected death in infancy

References

1. Hardelid P, Dattani N, Cortina-Borja M, Gilbert R. Contribution of respiratory tract infections to child deaths: a data linkage study. *BMC Public Health* 2014; **14**: 1191.

2. Taylor BJ, Garstang J, Engelberts A, et al. International comparison of sudden unexpected death in infancy rates using a newly proposed set of cause-of-death codes. *Arch Dis Child* 2015; **100**(11): 1018-23.
